# The Utility of Amino Acid Metabolites in the Diagnosis of Major Depressive Disorder and Correlations with Depression Severity

**DOI:** 10.3390/ijms24032231

**Published:** 2023-01-23

**Authors:** Cyrus Su Hui Ho, Gabrielle Wann Nii Tay, Hai Ning Wee, Jianhong Ching

**Affiliations:** 1 Department of Psychological Medicine, Yong Loo Lin School of Medicine, National University of Singapore, Singapore 119074, Singapore; 2 Cardiovascular and Metabolic Disorders Programme, Duke-NUS Graduate Medical School, Singapore 169857, Singapore

**Keywords:** major depressive disorder, amino acids, diagnostic, depressive severity

## Abstract

Major depressive disorder (MDD) is a highly prevalent and disabling condition with a high disease burden. There are currently no validated biomarkers for the diagnosis and treatment of MDD. This study assessed serum amino acid metabolite changes between MDD patients and healthy controls (HCs) and their association with disease severity and diagnostic utility. In total, 70 MDD patients and 70 HCs matched in age, gender, and ethnicity were recruited for the study. For amino acid profiling, serum samples were analysed and quantified by liquid chromatography-mass spectrometry (LC-MS). Receiver-operating characteristic (ROC) curves were used to classify putative candidate biomarkers. MDD patients had significantly higher serum levels of glutamic acid, aspartic acid and glycine but lower levels of 3-Hydroxykynurenine; glutamic acid and phenylalanine levels also correlated with depression severity. Combining these four metabolites allowed for accurate discrimination of MDD patients and HCs, with 65.7% of depressed patients and 62.9% of HCs correctly classified. Glutamic acid, aspartic acid, glycine and 3-Hydroxykynurenine may serve as potential diagnostic biomarkers, whereas glutamic acid and phenylalanine may be markers for depression severity. To elucidate the association between these indicators and clinical features, it is necessary to conduct additional studies with larger sample sizes that involve a spectrum of depressive symptomatology.

## 1. Introduction

Metabolomics is the terminus of the omics cascade that identifies and quantifies small molecules, known as metabolites, which are the end and by-products of complex biosynthetic and catabolism pathways [1]. As indicators of normal biological and pathological processes, metabolites include amino acids, nucleic acids, carbohydrates, fatty acids, cholesterol, and organic acids that may be objectively measured and analysed. They reflect the interaction of genomics, proteomics, and environmental factors and provide dynamic profiling of the individual’s internal state. Metabolites operate as direct markers of biochemical activity and, hence, most closely correlate with the observed phenotype compared to genes and proteins, which are modified by epigenetic and post-translational changes. Thus, metabolomics could hold higher potential than other omics methods for discovering illness-specific biological signatures [2,3].

Within the metabolome, amino acids have been regarded as potential biomarkers for depression as they are involved in homeostatic responses and downstream signalling pathways that are pivotal in the pathomechanisms of depressive disorders [4,5]. The well-established monoamine hypothesis postulates that the deficiency of monoamine neurotransmitters, such as serotonin, dopamine, and norepinephrine, contributes to depression. This was supported by antidepressants targeting monoamine depletion to increase the levels of neurotransmitters, thereby enhancing monoaminergic system function [6]. Furthermore, serotonin and norepinephrine are synthesised from their amino acid precursors, tryptophan and tyrosine, respectively, and lowered levels of these amino acids have been reported in depressed patients [7]. When supplemented into the diet, amino acids are converted to neurotransmitters and can also alleviate depressive symptoms [8]. Specialised carrier-mediated mechanisms allow centrally located amino acids to traverse the blood–brain barrier (BBB), making them available for measurement in peripheral blood.

Alterations in the peripheral amino acids are shown to discern between depressed and healthy persons with high reliability, rending them worthwhile candidates for illness predictions and elucidating underlying aetiology of depression and symptomatology. In depressed subjects, studies have found that levels of glutamate, aspartate, alanine, taurine, glycine, and gamma-aminobutyric acid (GABA) are increased, while levels of lactate, tryptophan, tyrosine, valine, and dopamine are decreased [1]. However, the discrepancy in the direction of change in amino acid levels for major depressive disorder (MDD) patients exists across multiple studies [9]. This could be explained by factors such as small sample sizes with limited statistical power, the failure to account for confounding variables such as age, gender, and nutritional status, and the inability to compensate for multiple testing.

As a panel, GABA, dopamine, kynurenine, and tyramine was found to accurately differentiate depressed subjects from healthy subjects at approximately 96.8% and 95.3% in the training and testing set, respectively, and precisely separate subjects with unipolar and bipolar depression [10]. In another study, branched-chain amino acids (BCAA) isoleucine, leucine and valine decreased considerably in depressed individuals, with the concentration of these amino acids inversely associated with the Hamilton Depression Rating Scale (HAM-D) scores. The reduced BCAA possibly contributed to depression by inhibiting the mammalian target of rapamycin (mTor), which reduces protein synthesis underlying the neuroplasticity [11]. Amino acids and their metabolites may also correlate with depressive symptomatology. Kynurenine, 3-Hydroxykynurenine, and kynurenate were related to suicidal ideation and the severity of depression [12], suggesting their predictive potential in symptom manifestation and disease progression. Amino acids may also be used to predict treatment response. One study found a higher ratio of tyrosine/large neutral amino acids (LNAAs) and a lower ratio of tryptophan/LNAAs to predict a favourable response to serotonergic antidepressants [13]. Overall, amino acid metabolism holds the potential to clarify the pathophysiology of MDD and facilitate the prediction of therapy response.

In sample collection, serum and plasma have been regarded as more valuable biofluids than cerebrospinal fluid (CSF), because they are easily accessible and may be collected using standard operating procedures [14]. Additionally, roughly 80% of the proteins detected in CSF originate from the blood via the blood–CSF barrier, with only 20% coming from the brain [15]. Therefore, examining blood for depression biomarkers can be justified, particularly given the many linkages between the brain and periphery involving inflammatory and neuroendocrine pathways [16].

This study hypothesised that MDD patients had varying concentration levels of amino acid compared to healthy controls (HCs) and could be used as diagnostic markers for MDD. The first aim was to determine which amino acids differed in concentrations between MDD patients and HCs in the Singapore setting and how they correlated with the severity of depression and other clinical parameters. The second aim was to ascertain the sensitivity and specificity of using specific amino acid metabolites to classify patients with MDD and HCs.

## 2. Results

### 2.1. Demographic and Clinical Characteristics of HCs and MDD Patients

There were no significant differences in age, sex, or ethnicity between the HCs and MDD patients (*p* > 0.05; see Table 1). However, there were significant differences between the two groups regarding years of education, the prevalence of mental illness in the family, history of trauma, perceived social support, and scores on the HAM-D. MDD patients were found to have lower levels of education than HCs (*p* < 0.001). They were also more likely to have a history of mental illness in their families (*p* = 0.032), experienced trauma (*p* < 0.001), and reported lower social support (*p* < 0.001) than HCs. On the HAM-D, as predicted, depressed individuals scored much higher than HCs (*p* < 0.001), with 70% having moderate to severe depression. In total, 77.1% of depressed individuals had never been hospitalised. Overall, 85.7% of patients (n = 60) were on pharmacotherapy, with most receiving antidepressants and some taking antipsychotics, anxiolytics, sedatives, or mood stabilisers. The average antidepressant dose of patients was approximately 33 mg of fluoxetine per day, a moderate dose for treating depression.

### 2.2. Amino Acid and Kynurenine Pathway Metabolite Concentrations

**Figure 1:**
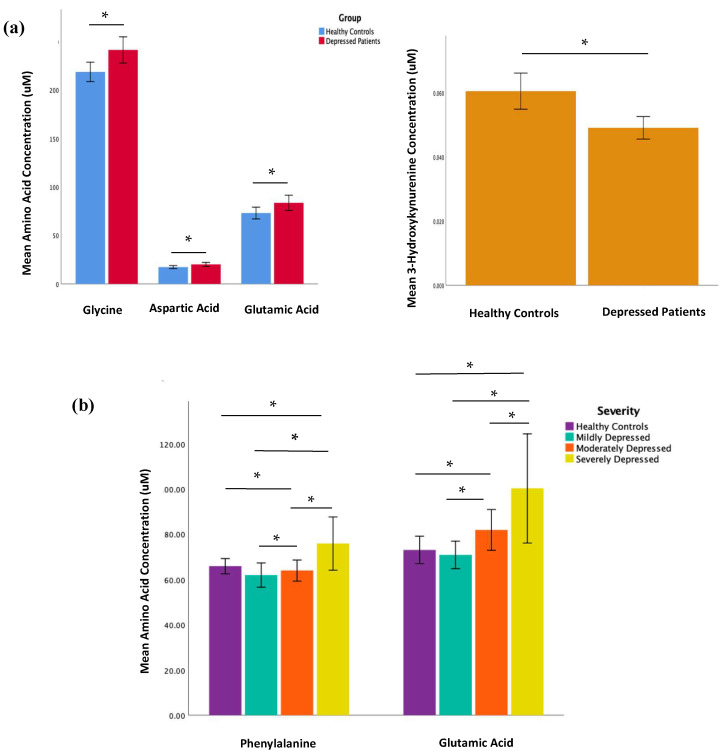
Comparison of mean metabolite concentrations in blood serum of (**a**) HCs (n = 70) and MDD patients (n = 70) and (**b**) HCs (n = 70) alongside MDD patients with differing disease severity (n = 21 mild MDD; n = 30 moderate MDD; n = 19 severe MDD) * *p* value ≤ 0.05.

**Figure 2:**
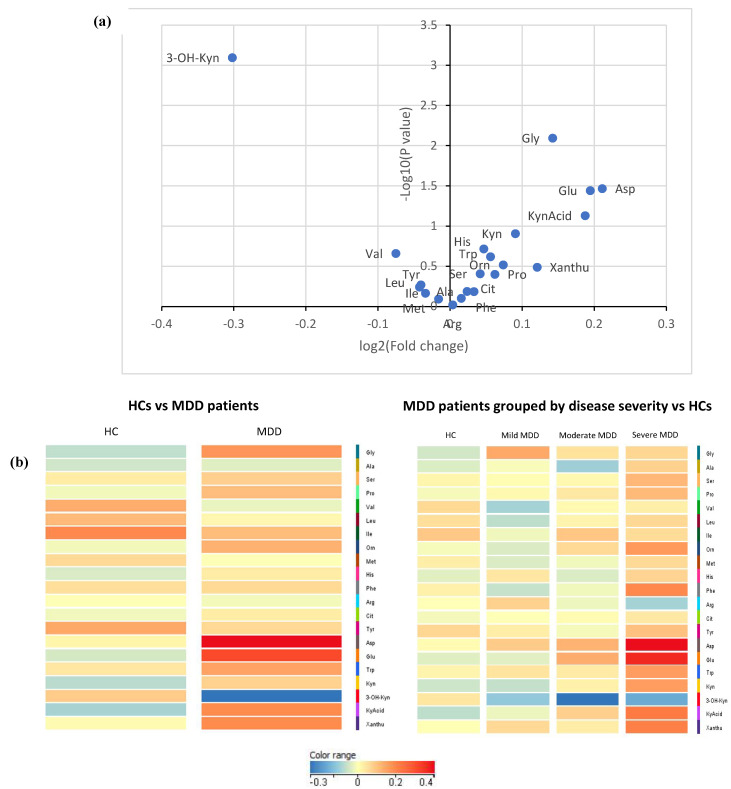
(**a**) Volcano plot comparing metabolite concentrations between MDD patients (n = 70) and HCs (n = 70). A larger difference in concentration for a particular amino acid would result in a higher log_2_ (fold change) value, and a point that lies more towards the right side of the x-axis. The more significant the difference in concentration, the smaller the *p* value and, resultingly, the higher the log_10_ (*p* value). (**b**) Heat maps comparing metabolites between MDD patients (n = 70) and HCs (n = 70). Colour intensity represents fold change values, with red representing an increase and blue a decrease.

Statistically, MDD patients had considerably higher concentrations of glycine, aspartic acid, and glutamic acid but a lower concentration of 3-Hydroxykynurenine (all *p* < 0.05) than HCs (see Table 2; Figure 1a); none of these differences in metabolite concentration, however, survived the Bonferroni correction for multiple comparisons. In comparing MDD patients of differing disease severity, phenylalanine and glutamic acid concentrations increased with increasing depression severity (see Table 3; Figure 1b); similar to the comparisons conducted between HCs and MDD patients, most significant differences in metabolite concentrations did not survive the Bonferroni correction—with the exception of glutamic acid. There was no measurable statistical difference in metabolite levels between unmedicated patients and matched HCs or between medicated and unmedicated patients. However, the sample size of unmedicated patients (n = 10) is too small to draw definitive conclusions. No significant correlations were found between the metabolites, clinical characteristics (e.g., past trauma, family history of psychiatric illness, age of onset of depression, depression duration, history of suicide, psychiatric admission), and depressive symptoms. A volcano plot visually demonstrates the relationship between the *p* value and magnitude of the difference in metabolite concentrations between MDD patients and HCs (see Figure 2a). Visual representations of the fold change difference in metabolites between HCs and MDD patients and between depression severity are shown as heatmaps in Figure 2b. The intensity of the colour denotes the fold change, with red representing an increase and blue a decrease. MDD patients generally had more metabolites with increased fold change than HCs; this trend was also positively associated with depression severity.

### 2.3. Differentiating MDD from HC Using Metabolites

**Figure 3:**
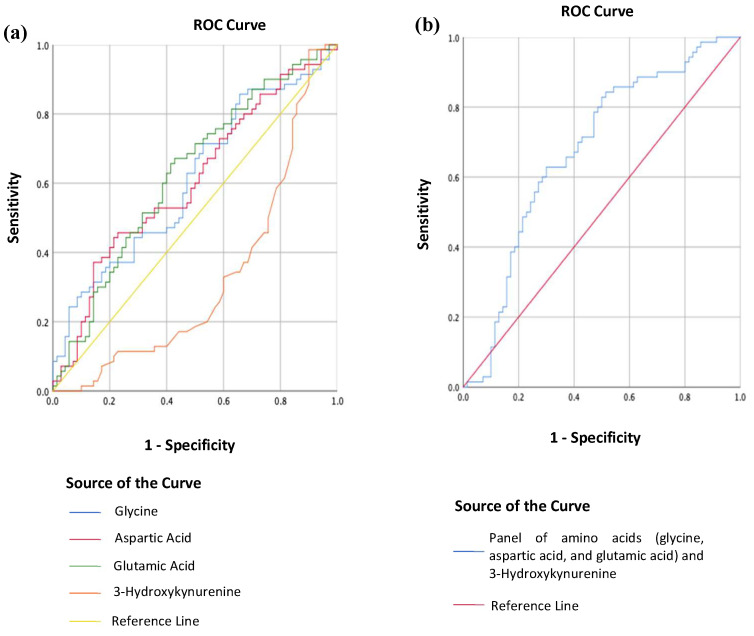
(**a**) Receiver operating curve (ROC) analysis of the individual metabolites’ concentrations in blood serum between MDD patients (n = 70) and HCs (n = 70); (**b**) Receiver operating curve (ROC) analysis of a combination of glycine, aspartic acid, glutamic acid, and 3-Hydroxykynurenine concentrations in blood serum between MDD patients (n = 70) and HCs (n = 70).

MDD patients could be distinguished from HCs using individual concentrations in blood serum of glycine, aspartic acid, glutamic acid, and 3-Hydroxykynurenine (see Figure 3a). The area under the ROC curve (AUC) was 0.61 [95% CI, (0.52, 0.70)], 0.61 [95% CI, (0.51, 0.70)], and 0.63 [95% CI, (0.54, 0.72)] for glycine, aspartic acid, and glutamic acid respectively. An optimal glycine concentration of 215.52 uM correctly classified 62.9% of MDD patients (proportion of patients/measurements: 44/70) and 54.1% of HCs (proportion of controls/measurements: 37/70; positive predictive value (PPV) = 0.57; negative predictive value (NPV) = 0.59), while an optimal aspartic acid concentration of 16.03 uM correctly classified 58.6% of MDD patients (proportion of patients/measurements = 41/70) and 51.4% of HCs (proportion of controls/measurements: 36/70; PPV = 0.55; NPV = 0.55). An optimal glutamic acid concentration of 66.94 uM correctly classified 68.6% of MDD patients (proportion of patients/measurements: 48/70) and 52.9% of HCs (proportion of controls/measurements: 37/70; PPV = 0.593; NPV = 0.627). The AUC was 0.32 [95% CI, (0.23, 0.42)] for 3-Hydroxykynurenine.

However, using a combination of glycine, aspartic acid, glutamic acid, and 3-Hydroxykynurenine concentrations in blood serum, patients with MDD could be discriminated from HCs with enhanced accuracy (see Figure 3b). The AUC was 0.68 [95% CI, (0.59, 0.77)], and an optimal threshold value of 0.51 correctly classified 65.7% of MDD patients (proportion of patients/measurements: 47/70) and 62.9% of HCs (proportion of controls/measurements: 44/70; PPV = 0.64; NPV = 0.65).

## 3. Discussion

### 3.1. Glutamic Acid, Aspartic Acid, Glycine, and Phenylalanine in Depression

The key findings from the amino acid analysis are (i) MDD patients had significantly higher levels of glutamic acid, aspartic acid, and glycine than HCs; (ii) phenylalanine and glutamic acid significantly differentiated MDD patients with varying severity, with higher levels corresponding to more severe depression scores.

However, research on amino acid levels in depression has been conflicting thus far. According to the glutaminergic hypothesis of depression, the pathophysiology of depression relates to anomalies of glutamatergic neurotransmission; specifically, a deficiency of glutamate with the loss of glutamatergic neurons in the orbitofrontal cortex. Nevertheless, this study indicated that depressed patients had higher glutamate levels, which defied this notion but was consistent with several other studies [9,17,18,19]. The significant positive association between depression severity and glutamate levels observed in this study has also been observed in prior research [18]. Additionally, post-mortem investigations have revealed elevated levels of glutamate in the medial prefrontal cortex of depressed patients relative to healthy controls [20]. According to a meta-analysis based on pooled proton magnetic resonance spectroscopy findings, the medial frontal cortex of depressed patients had lower levels of glutamatergic metabolites [21]. However, another meta-analysis found peripheral blood glutamate levels to be considerably higher in depressed patients than in controls, though the studies were highly heterogeneous [22]. Inconsistent results have been attributed to the clinical and biochemical variability of depression, methodological inconsistency, and the possibility that the complex pathophysiology of depression entails altered neurotransmission and signalling, which are more than just a simple regulation of glutamate. Similarly, our findings of increased aspartic acid levels in depressed patients corroborated two studies, albeit three others revealed no significant changes. As for glycine, our study’s finding of increased glycine levels in MDD patients relative to HCs corroborated the results of two other investigations [18,23]; however, other studies have reported no significant differences [9,17]. Serum glycine levels were also related to suicidal ideation, with levels significantly higher in veterans expressing suicide ideation than those who did not report suicidal thoughts [23].

Increased glutamate, aspartate and glycine levels may contribute to depression via their action as N-methyl-D-aspartate (NMDA) receptor agonists [24]. In the CNS, glutamate and aspartate are the two principal excitatory neurotransmitters, with glutamatergic neurons constituting around 80% of neocortical synapses. The NMDA receptor is an ionotropic glutamate receptor (the other two being α-amino-3-hydroxy-5-methyl-4-isoxazolepropionic acid (AMPA) and kainite receptors) with high calcium permeability that is activated primarily by glutamate and aspartate, with glycine serving as a co-agonist. The interaction of two glutamate or aspartate molecules and two glycine molecules is required for NMDA receptor activation. Glutamate and aspartate bind to the glutamate-binding site, albeit aspartate activates the receptors less potently than glutamate, whereas glycine attaches to the glycine-binding site. These three amino acids cross the BBB via specific transport systems into the CNS [25], which may activate various neuronal receptors, including the NMDA receptors.

NMDA receptors are ubiquitous in the cortical brain regions and hippocampus and play a pivotal role in several biological processes. They are necessary for developing the CNS and influence mechanisms underlying learning, memory, and neuroplasticity [26]. Hypo-functioning NMDA receptors can lead to cognitive deficits, whereas excessive activation induces excitotoxicity and neurodegeneration. In addition, calcium influx increases when NMDA receptors are overactive, leading to intracellular calcium excess. The calcium overload causes malfunction of the mitochondria with enhanced oxidative stress from increased reactive oxygen species, which triggers pro-inflammatory pathways and gives rise to tissue/organ damage [27]. Consequently, aberrant levels of NMDA receptor expression and function have been associated with neuropsychiatric disorders, such as stroke, hypoxia-ischemia, Parkinson’s disease, epilepsy, and Alzheimer’s disease, along with mood disorders and schizophrenia [24].

NMDA receptors have a crucial role in depression pathophysiology, as evidenced by elevated amino acids, particularly glutamate and aspartate in patients, and the amelioration of symptoms following NMDA receptor inhibition [21]. NMDA receptor antagonists, such as ketamine, and novel drugs, such as rapastinel, apimostinel, 4-chlorokynurenine, and rislenemdaz, have demonstrated promising efficacy in treating MDD and treatment-resistant depression [28,29]. Traditionally, ketamine is believed to boost glutamate levels in depressed patients’ prefrontal cortex by blocking the NMDA receptors on GABAergic interneurons, which disinhibits afferents to the glutamatergic neurons, activating the AMPA receptors. This leads to a cascade of signalling changes, including eukaryotic elongation factor 2 kinase inhibition and activation of tropomyosin-related kinase B, BDNF, and mTOR, ultimately resulting in elevated synaptic protein levels in the prefrontal cortex [30,31]. However, in vivo micro-dialysis could not establish a link between higher glutamate levels and ketamine’s antidepressant effect, indicating the possible existence of alternative processes behind this effect [32]. Recent research found that ketamine inhibits neuronally produced glutamate through retrograde activation of presynaptic adenosine A1 receptors, which may have contributed to its rapid antidepressant effect [33]. These findings support the glutamate theory of depression, which is based on abnormal glutamate neurotransmission and fill in the gaps left by the monoamine theory’s shortcomings, such as delayed medication effects despite normalised monoamine levels, inadequate drug response, and the increase in treatment-resistant depression [34]. Modifying glutaminergic systems may be an alternative to conventional serotonin and dopamine systems, and thus, having significant implications for depression treatment.

Compared to the other three amino acids, the evidence regarding phenylalanine’s role in depression is less conclusive. According to the literature, the effect of increased phenylalanine in the blood on the CNS is primarily studied in patients with phenylketonuria [35]. Phenylalanine is catabolised to tyrosine as the primary pathway. However, the catabolism of phenylalanine to tyrosine is hampered in patients with phenylketonuria due to a deficiency of the phenylalanine hydroxylase (PAH) enzyme, causing an accumulation of phenylalanine in the blood (hyperphenylalaninemia).

While patients with phenylketonuria can still acquire adequate tyrosine from their diet, the excess phenylalanine has been found to have a long-lasting detrimental impact on brain function. Depression, anxiety, and psychosis are much more prevalent in phenylketonuria [36,37]. Phenylalanine has been shown in vitro to decrease synaptic density and cause synaptic impairments in primary rat hippocampus neurons [38], potentially via inducing oxidative stress [39,40]. Furthermore, phenylalanine and its metabolites block enzymes in the glycolysis pathway in brain tissues [38,41]. Additionally, because phenylalanine utilises the same amino acid transport system as other LNAAs (e.g., tryptophan, tyrosine, and BCAA), it is suggested that an excess of phenylalanine in the blood may compete for transport across the BBB, decreasing the availability of tryptophan, tyrosine, and BCAAs in the brain [35,36]. Moreover, phenylalanine may competitively inhibit the functioning of tyrosine hydroxylase (TH) and tryptophan hydroxylase (TPH) in the brain, resulting in shortages of dopamine and, particularly, serotonin. This mechanism is likely related to the high occurrence of mood disorders and anxiety in people with hyperphenylalaninaemia. Lastly, tetrahydrobiopterin (BH_4_) is a cofactor essential for the efficient functioning of phenylalanine hydroxylase. The BH_4_ level was shown to be lower in depressed patients, which could lead to PAH deficiency, limiting the transformation of phenylalanine to tyrosine and decreasing epinephrine, norepinephrine, and dopamine levels [42].

### 3.2. 3-Hydroxykynurenine in Depression

In this study, it was surprising to find that MDD patients had lower 3-Hydroxykynurenine levels than HCs, which contradicts findings from previous studies [43]. Regarding increased inflammation, it is theorised that the kynurenine pathway is triggered, and available tryptophan is shunted away from serotonin production and into the further breakdown, leading to an increase of the 3-Hydroxykynurenine output. 3-Hydroxykynurenine is commonly considered neurotoxic due to its ability to generate reactive oxygen species, alter mitochondrial function, and induce DNA damage [44], which could be related to depression. Nonetheless, studies reveal that 3-Hydroxykynurenine may possess antioxidant and scavenging characteristics that could be protective under specific conditions [45]. 3-Hydroxykynurenine inhibits lipid oxidation, protein oxidation, and nitration. Additionally, it can react with nitric oxide and serves as an effective nitric oxide scavenger. Some researchers have proposed that 3-Hydroxykynurenine functions more like a redox modulator than a neurotoxin [46] because it can exert both pro- and antioxidant effects in rat striatal tissue under different situations. However, when it generates oxidative stress, this action does not result in cell damage. Thus, lowered levels of 3-Hydroxykynurenine, as shown in this study, may also indicate diminished antioxidant capacity, which raises the likelihood of contracting depression. A meta-analysis of 101 human observational studies revealed wide variance in the direction of kynurenine pathway metabolites, with some research showing contradictory results [47]. In depressed patients, 3-Hydroxykynurenine levels have been observed to be greater [48], lower [49], or unchanged [50].

### 3.3. Using Metabolites to Differentiate MDD Patients from HCs

In this study, the AUC for glycine, aspartic acid, and glutamic acid were 0.61, 0.61, and 0.63, respectively. Although these three amino acids can discern between people with and without MDD, their ability falls short of clinically acceptable. By convention, an AUC value of 0.5 implies that a diagnostic test is incapable of distinguishing between the presence and absence of a condition; an AUC value ranging between 0.7 and 0.8 is good, 0.8 and 0.9 is outstanding, and any value above 0.9 indicates that the test has an exceptional discriminative ability [51]. Whilst the AUC values obtained in this study are not clinically robust enough, they lie close to the clinically acceptable range of 0.7 to 0.8, alluding to the possibility of using the aforementioned amino acids as biomarkers for MDD. 3-Hydroxykynurenine alone performs worse than a random classifier for MDD (AUC 0.32). One possible hypothesis is that the kynurenine pathway (the principal route for tryptophan breakdown and, thus, 3-Hydroxykynurenine) may be more susceptible to antidepressant effects than the other amino acids [50]. Interestingly, when these three amino acids are combined with 3-Hydroxykynurenine as a panel, the AUC improves to 0.68 (i.e., approaching 0.7), with a sensitivity of 65.7% and a specificity of 62.9%, making this panel a suitable supplementation to existing diagnostic practice. As these results are based on a naturalistic study in which parameters such as blood collection time, medication intake, diet, and other lifestyle factors were not controlled, this panel of metabolites may have the prospects of easier adoption in clinical practice.

### 3.4. Strengths and Limitations

This study aimed to investigate the association of a broad range of amino acids with depression and identify metabolites that could be used as diagnostic markers. To our knowledge, this is the first of such studies in Singapore. The metabolite analysis should preferably extend beyond amino acids to facilitate the discovery of target markers. However, this study sought to identify a rapid, effective, and relatively inexpensive diagnostic approach capable of rapidly distinguishing MDD patients from healthy individuals to provide timely treatment with greater efficacy. Given the limited time available, multiple metabolite testing may not be feasible in the clinical setting. It could further discourage patients from consenting to the tests due to increased expenses and delays in receiving results.

Blood samples should preferably be taken in the morning, and food restrictions should be enforced one day before. This would be challenging and potentially hinder patients from getting tested. Furthermore, sample collection time or enforcing restrictions, such as fasting, has been demonstrated in studies not to affect the fluctuation in amino acid levels within 48 h preceding the blood collection [52]. In addition, not all MDD patients are medication naive, so the findings of our study could be attributed to treatment effects. Because this was a cross-sectional study, it is impossible to determine a causal relationship between depression onset and changes in amino acid concentrations. The absence of significant differences in almost all metabolite concentrations between groups upon correcting for multiple comparisons, as well as relationships between specific amino acids and clinical parameters, may have been due to the limited sample size with reduced power. Further research is required to evaluate the clinical usefulness of these biomarkers, preferably involving larger populations.

## 4. Materials and Methods

### 4.1. Sample Size

For a two-tailed Welch’s *t*-test to achieve 80% power and 0.05 probability of type I errors, our sample size was derived from the effect size of glutamic acid concentration (which demonstrated a Cohen’s d value of 0.91) based on the study by Pan and colleagues [10], which compared 50 healthy controls and 50 unmedicated MDD patients. This study showed that the minimum number of subjects needed to detect differences in amino acid concentrations between groups was 26 per group.

### 4.2. Participants

This cross-sectional study recruited 70 MDD patients and 70 HCs matched for age, sex, and ethnicity. All 140 participants were English-speaking, right-handed, and aged between 21 and 50 years. Patients were recruited from the outpatient psychiatry clinics of a university hospital in Singapore, where they had been diagnosed with MDD by a psychiatrist in accordance with the criteria in the fifth edition of the Diagnostic and Statistical Manual of Mental Disorders (DSM-5). Patients with other significant psychiatric disorder comorbidities, such as schizophrenia, bipolar depression, and substance use disorder, were excluded from the study. Healthy individuals were recruited from the general community by word-of-mouth and matched with MDD patients based on age, sex, and ethnicity. Individuals were excluded if they had conditions that could affect the central nervous system, including cerebrovascular diseases, respiratory diseases, hepatic diseases, kidney diseases, cancer, epilepsy, or intellectual disability, as well as a history of psychiatric and/or neurological disorders. Subjects deemed qualified for the trial were promptly recruited. Each patient’s depressive symptoms and disease severity were assessed using the 21-item Hamilton Rating Scale for Depression (HAM-D 21).

All study protocols adhered to the Helsinki Declaration’s ethical standards and the Belmont Report’s ethical principles. The Domain Specific Review Board of the National Healthcare Group, Singapore, approved the study (protocol number 2019/00141). The specifics of the study were presented to every individual, and their written, informed consent was obtained. All information gathered was deidentified. All individuals provided their sociodemographic data, and patients’ clinical information was obtained from them and verified with computer records. Both groups of subjects completed written questionnaires and had their blood drawn during the study visit. As subjects’ samples were collected once they enrolled on the study, it was difficult to standardise the collection time and lifestyle characteristics, such as the subjects’ nutrition and sleep.

### 4.3. Blood Collection and Metabolite Analysis

Serum amino acid profiling was conducted using procedures previously employed in other studies [53]. A total of 21 metabolites were analysed: 17 from the preset amino acid panel (glycine, alanine, serine, proline, valine, leucine, isoleucine, methionine, histidine, phenylalanine, tyrosine, aspartic acid, glutamic acid, ornithine, citrulline, arginine, and tryptophan) and four from the preset kynurenine panel (kynurenine, kynurenic acid, xanthurenic, and 3-Hydroxykynurenine) provided by the Metabolomics Facility. Blood samples were collected from subjects and handled within an hour of extraction. After clot formation, the samples were centrifuged for a 10-min duration at a temperature of 4 °C, with a g-force of 2000. This produced serum samples, which were kept at −80 °C and transported to the laboratory for analysis using dry ice. Methanol was used to extract 50 uL of serum samples, which were then dried in the presence of nitrogen gas. For the liquid chromatography-mass spectrometry (LC-MS) analysis, the dried extracts were first derivatised with 3 mL of hydrochloric acid in butanol and then diluted with water. The deuterated stable isotopes of the various amino acids were utilised as internal standards in this targeted LC-MS experiment. The LC-MS analysis was carried out using a quadrupole-ion trap mass spectrometer (QTRAP 5500, AB Sciex, Olympia, DC, USA) in conjunction with an Agilent 1290 Infinity liquid chromatography system (Agilent Technologies, Santa Clara, CA, USA). A C18 column (Phenomenex, 100 × 2.1 mm, 1.6 m, Luna^Ⓡ^ Omega, Torrance, CA, USA) was used to separate the samples. Chromatography separation was performed using Mobile phase A (Water) and Mobile phase B (Acetonitrile), which contained 0.1% formic acid. The LC run was conducted at a 0.4 mL min^−1^ flow rate for 0.8 min with an initial gradient of 2% B, followed by increases to 15% B in 0.1 min, 20% B in 5.7 min, 50% B in 0.5 min, and 70% B in 0.5 min, after which there was a 0.9 min re-equilibration of the column to the initial run conditions (2% B). Electrospray ionisation was used to ionise all compounds in the positive mode. The MultiQuant^TM^ 3.0.3 software (AB Sciex, Olympia, DC, USA) was used to integrate the chromatograms. For absolute quantification of the amino acids, the ratios of the metabolites to their respective internal standards were compared to an external calibration curve comprising all reported amino acids.

### 4.4. Statistical Analysis

IBM SPSS Statistics 20.0 was used for all statistical analyses. The chi-square test and the independent *t*-test were used to compare categorical and continuous variables between groups. Analyses of the correlations between the various amino acids and clinical features were conducted with the Spearman’s and Pearson’s correlation for categorical and continuous variables, respectively. To determine the classification ability of the amino acid metabolite concentrations in distinguishing MDD patients from HCs, the classic receiver-operating characteristic (ROC) analysis was utilised. The likelihood test was used to construct the ROC curve, and the Youden’s Index was optimised to get the appropriate cut-off point for the calculated probability. All tests had a significance level of 0.05 and were two-tailed.

## 5. Conclusions

The concentration levels of glutamic acid, aspartic acid and glycine are higher in clinically depressed individuals. Conversely, 3-Hydroxykynurenine is lower in depressed people and may serve as potential diagnostic biomarkers, while glutamic acid and phenylalanine may act as markers for depression severity. Future research with bigger sample size, including a range of depressive symptomatology, may be necessary to clarify the relationship between these markers and clinical characteristics.

## Figures and Tables

**Table 1:** Demographic and clinical characteristics of HCs and MDD patients.

	HC (n = 70)	MDD (n = 70)	*p* Value
**Age (years)**	28.2 (SD 7.3)	28.3 (SD 7.2)	0.926
**Sex**			1.000
**Male**	16 (22.9%)	16 (22.9%)	
**Female**	54 (77.1%)	54 (77.1%)	
**Ethnicity**			1.000
**Chinese**	45 (64.3%)	45 (64.3%)	
**Malay**	15 (21.4%)	15 (21.4%)	
**Indian**	9 (12.9%)	9 (12.9%)	
**Eurasian**	1 (1.4%)	1 (1.4%)	
**Education (years)**	15.6 (SD 1.2)	14.5 (SD 1.8)	<0.001
**HAM-D**	1.9 (SD 2.5)	19.8 (SD 5.4)	<0.001
**Mild (8–16)**	4 (5.7%)	21 (30.0%)	
**Moderate (17–23)**	0	30 (42.9%)	
**Severe (≥24)**	0	19 (27.1%)	
**Family psychiatric history**	17 (24.3%)	30 (42.9%)	0.032
**History of trauma**	14 (20.0%)	35 (50%)	<0.001
**Age at MDD onset (years)**		20.7 (SD 7.5)	
**Duration of illness (years)**		7.9 (SD 6.5)	
**Past admission to psychiatric ward**		16 (22.9%)	
**Past suicide attempt**		32 (45.7%)	
**Perceived social support**			<0.001
**Poor**	0	17 (24.3%)	
**Average**	18 (25.7%)	44 (62.9%)	
**Good**	52 (74.3%)	9 (12.9%)	
**Pharmacotherapy**		60 (85.7%)	
**Antidepressants**		58 (96.7%)	
**Antipsychotics**		10 (16.7%)	
**Anxiolytics and sedatives**		8 (13.3%)	
**Mood stabilisers**		4 (6.7%)	
**Fluoxetine equivalent dose (mg/day)**		32.79 (SD 2.5)	
**Diazepam equivalent dose (mg/day)**		8.75 (SD 1.8)	
**Chlorpromazine equivalent dose (mg/day)**		91.21 (SD 20.6)	

**Table 2:** Differences in mean metabolite concentrations of blood serum of HCs and MDD patients.

Metabolite	Concentration (uM)				
	HCs (n =70)	MDD (n = 70)	*t* statistic	*p* Value	Corrected *p* Value (Bonferroni)
Glycine	218.5 (SD 42.1)	241.2 (SD 56.7)	−2.68	0.008	0.168
Alanine	427.6 (SD 81.9)	434.6 (SD 99.1)	−0.455	0.650	1.000
Serine	130.6 (SD 19.8)	134.4 (SD 31.2)	−0.862	0.391	1.000
Proline	193.3 (SD 59.2)	201.8 (SD 59.1)	−0.849	0.397	1.000
Valine	253.1 (SD 61.7)	240.2 (SD 61.7)	1.24	0.219	1.000
Leucine	123.7 (SD 38.5)	120.1 (SD 36.8)	0.567	0.572	1.000
Isoleucine	69.4 (SD 24.2)	67.7 (SD 22.8)	0.408	0.684	1.000
Ornithine	69.1 (SD 20.0)	72.7 (SD 21.4)	−1.03	0.303	1.000
Methionine	25.8 (SD 6.6)	25.5 (SD 7.4)	0.242	0.809	1.000
Histidine	77.4 (SD 11.1)	80.0 (SD 11.9)	−1.31	0.192	1.000
Phenylalanine	65.9 (SD 14.0)	66.6 (SD 17.1)	−0.27	0.789	1.000
Arginine	101.5 (SD 23.2)	101.7 (SD 27.3)	−0.057	0.954	1.000
Citrulline	27.2 (SD 7.5)	27.8 (SD 9.0)	−0.451	0.653	1.000
Tyrosine	65.4 (SD 18.2)	63.6 (SD 16.6)	0.615	0.539	1.000
Aspartic Acid	17.4 (SD 6.4)	20.1 (SD 8.6)	−2.14	0.034	0.714
Glutamic Acid	73.0 (SD 25.5)	83.6 (SD 32.9)	−2.12	0.036	0.756
Tryptophan	50.9 (SD 9.8)	52.9 (SD 10.4)	−1.18	0.240	1.000
Kynurenine	1.27 (SD 0.294)	1.35 (SD 0.330)	−1.55	0.124	1.000
3-Hydroxykynurenine	0.061 (SD 0.024)	0.049 (SD 0.014)	3.43	<0.01	0.210
Kynurenic acid	0.037 (SD 0.014)	0.042 (SD 0.019)	−1.80	0.074	1.000
Xanthurenic acid	0.017 (SD 0.008)	0.018 (SD 0.009)	−0.99	0.324	1.000

**Table 3:** Differences in mean metabolite concentrations of blood serum of patients with differing MDD severities.

	Concentration (uM)					
Metabolite	Mild MDD (n = 21)	Moderate MDD (n = 30)	Severe MDD (n = 19)	*F* Statistic	*p* Value	Corrected *p* Value (Bonferroni)
Glycine	249.9 (SD 49.8)	235.3 (SD 55.0)	240.8 (SD 67.3)	0.399	0.672	1.00
Alanine	438.3 (SD 100.5)	414.6 (SD 105.9)	462.2 (SD 83.0)	1.38	0.259	0.313
Serine	129.0 (SD 16.3)	132.3 (SD 31.4)	143.8 (SD 41.5)	1.24	0.295	0.419
Proline	199.8 (SD 76.6)	199.2 (SD 53.3)	208.1 (SD 46.9)	0.148	0.863	1.00
Valine	225.3 (SD 45.3)	247.8 (SD 77.1)	244.8 (SD 48.6)	0.887	0.417	1.00
Leucine	113.3 (SD 32.4)	121.4 (SD 38.1)	125.4 (SD 40.0)	0.564	0.571	1.00
Isoleucine	64.1 (SD 20.8)	70.6 (SD 26.2)	67.2 (SD 19.6)	0.506	0.605	1.00
Ornithine	67.1 (SD 17.3)	73.0 (SD 22.0)	78.5 (SD 24.0)	1.42	0.249	0.292
Methionine	24.7 (SD 7.0)	25.5 (SD 8.4)	26.5 (SD 6.6)	0.261	0.771	1.00
Histidine	81.1 (SD 9.5)	77.1 (SD 11.9)	83.3 (SD 13.5)	1.79	0.175	0.222
Phenylalanine	62.0 (SD 11.7)	64.0 (SD 12.4)	75.9 (SD 24.5)	4.28	**0.018**	0.134
Arginine	106.2 (SD 19.2)	101.2 (SD 28.1)	97.6 (SD 33.7)	0.493	0.613	1.00
Citrulline	27.1 (SD 6.14)	28.3 (SD 11.8)	27.9 (SD 6.5)	0.096	0.908	1.00
Tyrosine	63.2 (SD 15.1)	61.6 (SD 15.9)	67.2 (SD 19.6)	0.663	0.519	1.00
Aspartic Acid	18.1 (SD 5.1)	19.0 (SD 6.9)	24.1 (SD 12.5)	2.99	0.057	0.131
Glutamic Acid	70.9 (SD 13.4)	81.9 (SD 24.2)	100.3 (SD 50.2)	4.47	**0.015**	**0.013**
Tryptophan	52.0 (SD 11.1)	51.5 (SD 9.9)	56.3 (SD 10.3)	1.37	0.261	1.00
Kynurenine	1.28 (SD 0.419)	1.33 (SD 0.252)	1.46 (SD 0.316)	0.867	0.178	0.227
3-Hydroxykynurenine	0.052 (SD 0.018)	0.047 (SD 0.013)	0.050 (SD 0.014)	1.77	0.425	1.00
Kynurenic acid	0.040 (SD 0.025)	0.041 (SD 0.016)	0.045 (SD 0.016)	0.355	0.703	1.00
Xanthurenic acid	0.018 (SD 0.010)	0.018 (SD 0.010)	0.020 (SD 0.010)	0.273	0.863	1.00

## Data Availability

The datasets used and/or analysed during the current study are available from the corresponding author on reasonable request.
